# From co-existence to collaboration: The need to move towards an inclusive (pluricultural) scabies management approach in Debre Elias (Ethiopia)

**DOI:** 10.1371/journal.pntd.0014773

**Published:** 2026-10-14

**Authors:** Dereje Wonde, Kibur Engdawork, Anne Roemer-Mahler, Getnet Tadele, Mei Trueba

**Affiliations:** 1 Department of Social Anthropology, Faculty of Social Sciences, Bahir Dar University, Bahir Dar, Ethiopia; 2 Department of Global Health, Brighton and Sussex Medical School (BSMS), University of Sussex and University of Brighton, Brighton, United Kingdom; 3 Department of Sociology and Social Anthropology, College of Social Sciences, Arts and Humanities, Addis Ababa University, Addis Ababa, Ethiopia; 4 School of Global Studies, University of Sussex, Brighton, United Kingdom; Centers for Disease Control and Prevention, UNITED STATES OF AMERICA

## Abstract

Several large scabies outbreaks have occurred in the Amhara region of Ethiopia since 2015, and biomedical interventions focused on mass drug administration are often deployed to contain the disease. However, there is a persistent scabies incidence in the Amhara region. To understand this, we sought to explore how people living in Debre Elias understand and seek to manage scabies in their everyday lives. We collected data via a rapid ethnographic approach which included observations and semi-structured interviews. Fieldwork was conducted from February to July 2022 and involved 57 interviews with individuals who had experienced scabies and those who hadn’t but were at risk, as well as local health workers, traditional healers, and government officials. It also included participant and non-participant observation of persons with and without scabies in their homes and public spaces such as workplaces, social events, and treatment facilities. Critical analysis of scabies control and policy documents was also used to fully understand the local dynamics around scabies in Debre Elias. People living in Debre Elias commonly navigate both biomedical and traditional healing systems to manage various health problems, including scabies. However, this practice remains largely informal, as traditional healing is often marginalised in official scabies control strategies. Most individuals affected by scabies initially seek care from traditional healers, often turning to biomedical services only afterwards. Health-seeking and treatment decisions are shaped by the perceived causes of scabies, the cultural relevance and affordability of traditional medicine, and limited access to biomedical services. We conclude that traditional healing plays a central role in local scabies management. Recognising and engaging with existing medical pluralism may improve the acceptability, reach, and effectiveness of scabies programmes. Supporting pluralistic treatment pathways should therefore be considered an opportunity rather than a liability for scabies control.

## Author summary

Scabies remains a significant public health problem in the Amhara region of Ethiopia despite repeated control efforts, including mass drug administration campaigns. To better understand the frequent scabies occurrence, we explored how people in Debre Elias perceive and manage scabies in their everyday lives. Using a rapid ethnographic approach, we conducted interviews with community members, traditional healers, health workers, and government officials, alongside observations and analysis of local policy documents. We found that people commonly move between traditional and biomedical healthcare systems when seeking treatment for scabies. Most individuals initially consult traditional healers, often accessing biomedical services only later. Decisions about treatment are influenced by local understandings of scabies, the cultural relevance and affordability of traditional medicine, and challenges in accessing biomedical care. Despite its widespread use, traditional healing remains largely excluded from official scabies control strategies. Our findings show that traditional healing plays a central role in local scabies management. Scabies control programmes may be more effective if they recognise and engage with existing systems of traditional healing rather than focusing exclusively on biomedical interventions. Integrating and working alongside trusted local healing systems could improve the acceptability, reach, and sustainability of scabies control efforts.

## 1. Introduction

Globally, scabies affects more than 300 million people [1] with an estimated prevalence ranging from 0.2 to 71% [2,3]. Whilst in high-income countries sporadic cases of scabies occur in disadvantaged and institutionalized communities such as prisons, orphanages, and nursing homes for the elderly [4–6], community-level scabies is more frequent in low-income countries, with the highest prevalence occurring in impoverished, hot, and humid climates of the Pacific and Latin American regions [7]. A 2019 study in Ethiopia reported a 14.5% prevalence of scabies, with the Amhara region showing the highest rate at 19.6% compared to other regions in the country [8]. This region has experienced a large scale scabies outbreak since 2015, with a population prevalence ranging from 2% to 67% across the worst affected districts [9] and the latest documented prevalence being 13.4% of the population in 2021 [10]. Another outbreak occurred in the Debre Elias district in 2021. According to the 2021 annual scabies report by the Amhara Public Health Institute (APHI), a total of 4,247 scabies cases were reported in the Debre Elias district, representing the highest number of reported cases in the Amhara region [11].

Scabies prevention and control efforts in Ethiopia are largely informed by the WHO’s *Framework for Scabies Control*, which emerged from an informal expert consultation [12]. These recommendations primarily prioritise standardized biomedical interventions that can be implemented across diverse settings [12,13]. Key interventions include mass drug administration (MDA), clinic-based treatment using topical ointments following disease onset [14], and community-based strategies such as training Health Extension Workers (HEWs) to conduct scabies surveillance and deliver health education campaigns [15]. The 2015 large-scale scabies outbreak in the Amhara region marked a turning point in the institutionalisation of biomedical approaches as the primary strategy for scabies control approach in Ethiopia. The persistence of scabies across various districts in the Amhara region, however, suggests that current control efforts are insufficient.

Traditional healing practices are widespread in Ethiopia and continue to play a significant role in the country’s healthcare landscape, particularly in rural communities. Research indicates that nearly 80% of Ethiopians seek treatment via traditional healing practices [16]. Among others, this is due to the cultural competence of traditional healers, perceived efficacy, safety and affordability of traditional medicine, and lack of access to biomedical health services [16,17]. In line with the World Health Organization (WHO) definition, traditional medicine is understood in this paper as the health practices, approaches, knowledge, and beliefs that incorporate plant, animal, and mineral-based medicines, spiritual therapies, manual techniques, and exercises—applied individually or in combination—to diagnose, treat, and prevent illnesses or to maintain well-being [18]. Traditional medicine is frequently the preferred source of care for skin disorders in Ethiopia, owing to the accessibility and cultural competence of traditional healers, local explanatory models of disease causation, and the perceived efficacy of traditional remedies in treating dermatological conditions [17]. Nigussie for instance documented 52 types of local herbs used as common scabies treatments in peoples’ houses and in traditional treatment centres in Ethiopia [19].

Biomedical approaches to health and healing are often understood in contrast to traditional medicine. Grounded in scientifically oriented methods of diagnosis and treatment, biomedicine has become the dominant medical system in many contemporary societies, with state institutions, health policies, and biomedical professionals often positioning it as the primary source of legitimate medical knowledge and practice [20,21]. In contrast, traditional medicine commonly embraces holistic understandings of health that incorporate social, emotional, spiritual, and relational dimensions of wellbeing, as well as the specific problems individuals seek help for [22,23]. Biomedicine, by comparison, tends to conceptualise illness as residing primarily within the individual body and prioritises treatment through clinical and pharmaceutical interventions [20].

This study is motivated by the persistence of scabies in the Amhara region despite repeated biomedical control efforts often focused on mass drug administration campaigns. To better understand this persistence, we explored how residents of Debre Elias perceive and manage scabies, with particular attention to the implementation of biomedical interventions and their acceptance and uptake within the community. By examining how people navigate both biomedical and traditional healing systems, we also provide insights into the health-seeking practices and the sociocultural, political, and economic factors that shape treatment decisions. Our findings illuminate the limitations of predominantly biomedical and standardized approaches to scabies control and demonstrate the importance of engaging with existing forms of medical pluralism. In doing so, the study argues for more context-sensitive and culturally grounded scabies control strategies that recognise local therapeutic realities and integrate pluralistic treatment pathways, with implications not only for scabies management in Ethiopia but also for the control of other skin-related neglected tropical diseases in other settings.

## 2. Methods

### 2.1. Ethical considerations and approvals

Ethical approvals were obtained from the Brighton and Sussex Medical School (BSMS) Research Governance and Ethics Committee (RGEC) on 26 October 2021 (approval number ER/BSMS9K3J/1) and also from the Ethiopian Society of Sociologists, Social Workers, and Anthropologists (ESSSWA) on the 3^rd^ of November 2021 (protocol number 018/2021). To protect informants’ anonymity and confidentiality, in this paper we only use pseudonyms. Verbal informed consent was obtained from non-literate participants and written informed consent was obtained from literate participants. The principle of doing no harm to research participants was strictly adhered to during data collection. In addition, transport compensation was provided, and measures were taken to safeguard participants’ emotional wellbeing during interviews. Referral information was also shared with health workers for participants who were experiencing scabies at the time of the interview. All collected data were securely stored in the University of Sussex SharePoint repository to ensure confidentiality and prevent data loss. The study findings were presented to selected participants to obtain feedback and support respondent validation.

### 2.2. Research setting

This research was conducted in the Debre Elias district, located in the Amhara Region of north-western Ethiopia. Having daily temperatures of 18–27°C, an altitude ranging from 800-2,200 meters above sea level, and an average annual rainfall of 1,150 mm, Debre Elias has tropical conditions that are ideal for scabies and the mites that cause the disease. Debre Elias covers an area of approximately 94,000 hectares, with a population density ranging between 100 and 120 people per square kilometre [24]. It is divided to 21 *kebeles* (the lowest administrative unit in Ethiopia, equivalent to a village), four of which are urban and seventeen rural [25]. The capital of the district is also called Debre Elias and is a small town consisting of two *kebele* administrations. According to the 2007 population census, Debre Elias had a total population of 82,150, with 82.5% residing in rural areas and 17.5% in urban areas. The projected total population for Debre Elias in 2021 was 107,023 [26,27].

Debre Elias district is predominantly inhabited by Amharic-speaking people, with 99% adhering to Orthodox Christianity [24]. The remaining 1% are mostly Muslims, primarily residing in the urban *kebeles*. The social structure in the district is strongly influenced by religion, which significantly shapes the socio-economic life of the community as well as their health-seeking behaviours [28]. Healthcare services in Debre Elias are delivered through Ethiopia’s three-tier health system, comprising a primary hospital, health centres, and village level health posts staffed by Health Extension Workers (HEWs). Despite efforts to expand coverage, access to biomedical healthcare remains constrained for many residents by distance, transport costs, medicine shortages, and limited service availability [29].

### 2.3. Study design and data collection

Data was collected via a rapid ethnographic approach which included local document analysis, participant and non-participant observations, and semi-structured interviews. A rapid ethnographic approach (REA) is a qualitative research method that adapts traditional ethnography to short timelines, using a team-based, multi-method approach to quickly gather rich, contextual insights into a community’s behaviour and beliefs from an insider’s perspective [30]. The rapid ethnographic approach was chosen for this project to efficiently capture the complex cultural, social, and behavioural factors influencing how people in Debre Elias understood and managed scabies. This method allowed for in-depth qualitative insights and the holistic understanding of local health practices within a limited timeframe. The first author speaks the local language and is from this region which facilitated trust-building, engagement with potential research informants, and quick nuanced data collection.

Data were collected in Amharic by the first author between February and July 2022. Study participants included individuals who were experiencing scabies at the time of fieldwork, those who had previously experienced the disease, and those at risk of infection due to contact in living quarters. They were recruited from rural households, biomedical and traditional treatment facilities, and religious education centres. In addition, healthcare workers, Ministry of Health officials, and neglected tropical disease (NTD) experts were interviewed to gain insights into formal scabies control policies, management practices, and implementation strategies.

People with scabies and those at risk were recruited from five *kebeles*: Debre Elias town, *Degolma*, *Guay*, *Gofichma*, and *Dejiba*. These *kebeles* were purposively selected because they host the four health centers and the primary hospital in the district, making them important sites for examining both community experiences of scabies and treatment-seeking practices. Participants were recruited from various sites, including health posts, health centers, the primary hospital, holy water sites, religious education centers, and traditional herbal medicine centers. Nurses and health extension workers (HEWs), government-trained frontline health workers responsible for monitoring and responding to community health needs at the village level [31], assisted in identifying and approaching potential participants. Individuals who had scabies at the time of fieldwork or within the previous year, as identified through clinical diagnosis by health workers, were eligible for inclusion. In addition, individuals at risk of scabies —including the family members, friends and caregivers of those diagnosed with scabies — were recruited to explore the social dynamics associated with scabies and its management.

Participants were recruited using purposive and snowball techniques. With participants’ consent the interviews were audio recorded. Semi-structured interview guides were developed prior to data collection based on the study objectives and relevant literature on scabies perceptions, health-seeking practices, and disease management, and were approved by the research team and the relevant ethics committees (see supplementary information). During interviews, participants with experience of scabies were asked to explain their illness trajectories, including how they became affected by the condition and the ways in which they sought to prevent and treat it. Participants were also asked about their experiences of living with scabies, beliefs regarding its causes, perceptions of existing prevention and treatment strategies, and the factors influencing treatment-seeking decisions.

Both participant and non-participant observations were conducted in various settings frequented by people with scabies, including public spaces, homes, workplaces, social events, and treatment facilities. The first author accompanied informants during daily activities such as fetching water, working in agriculture, and visiting health centers for treatment. Observations were also made of healthcare provider–patient interactions at treatment facilities, as well as during mass drug administration (MDA) events and water, sanitation and hygiene WASH intervention visits conducted by health extension workers (HEWs) in the villages. Additionally, relevant documents—including brochures, government policies, and guidelines on scabies control and prevention—were reviewed and analysed to understand how scabies is managed within the government and healthcare system.

### 2.4. Data analysis

We analysed the data via both thematic and narrative analysis methods. Thematic analysis was used to identify, analyze and report patterns or themes within the data [32] whereas narrative analysis allowed the exploration of participant’s experience in relation to the research questions [33].

After preparation of interview and field observation data in text form, different levels and types of coding were employed. The first author carried out the coding and the remaining authors reviewed and suggested refinements when appropriate. We used hybrid coding involving both priori (deductive) and emerging (inductive) coding with words and short phrases to facilitate data analysis [34]. The next step was to look for patterns in the code list and identify crosscutting themes and sub-themes in the data. Analysis and interpretation of findings was informed by this coding activity. Parallel to this, by rereading the transcribed qualitative data, regularity, and contradictory explanations were identified, and finally each explanation was categorized under coherent thematic topics to generate meanings. Field notes from observations as well as document-based data were analyzed alongside the interview transcriptions to triangulate the evidence generated on the social dynamics of scabies. In sum, the data collected through different methods was analyzed concurrently to generate meanings and implications. NVivo 13 qualitative data management package was employed to facilitate the task of coding for data analysis.

## 3. Results

### 3.1. Participants characteristics

A total of 57 persons participated in the semi-structured interviews. Of these, 34 (59.7%) either had scabies or were at risk of scabies. Specifically, 23 (40.4%) had scabies at the time of fieldwork, and 11 (19.3%) were not experiencing scabies but were at risk - lived with a person affected by scabies, such as caregivers and religious students living in overcrowded places where scabies frequently occurred. The remaining 23 (40.3%) participants comprised government officials, health workers, NTDs experts, and traditional healers (see Fig 1).

**Fig 1:**
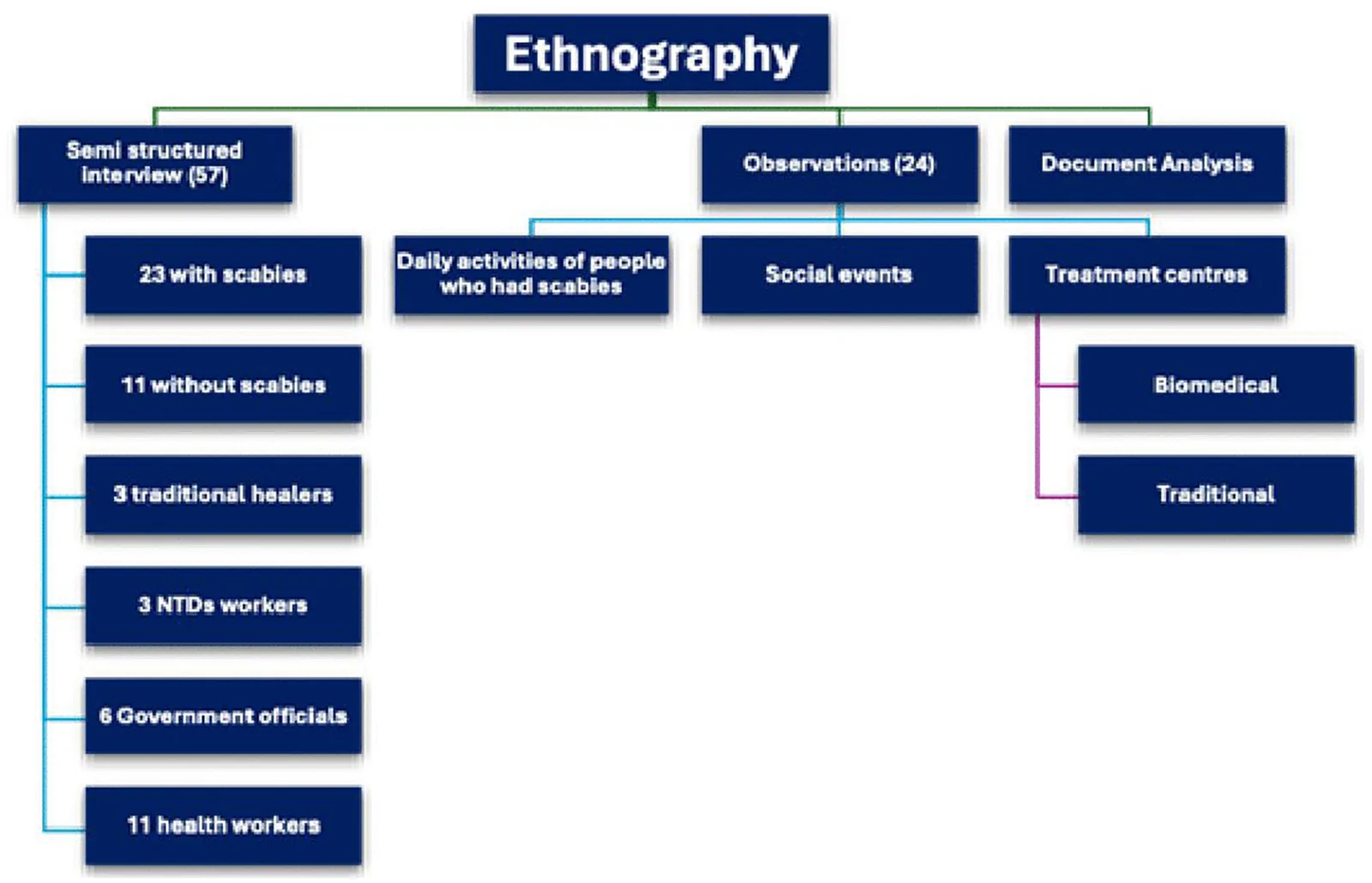
Methods of data collection and composition of informants.

The age of participants ranged from 18 to 60 years; 25 (43.9%) were female and 32 (56.1%) were male. 40 (70.2%) were married, 3 (5.3%) were divorced, and 14 (24.5%) were unmarried. Although children can also be affected by scabies those under the age of 18 were not included in this study, in accordance with the ethics approvals.

In terms of educational status, 12 (35.3%) of the participants with scabies or at risk of scabies were illiterate, while 24 (64.7%) were literate having received either religious education or formal education. Among the participants with scabies and at risk 24 (67.1%) were farmers. The largest family size reported by the participants was 11 people living in a single household.

### 3.2. Traditional etiological beliefs on scabies

Most people in Debre Elias attributed scabies to supernatural and personalistic causes, which in turn shaped people’s treatment-seeking journeys. For instance, scabies was attributed to divine sources among most Orthodox Christianity followers, and some individuals also stated that scabies outbreaks can occur as a punishment for people’s misdoings or sins:

*Realizing that many people were affected at the same time in the time of outbreak, some people in our village considered scabies outbreak as a sign of punishment from God.* (Yezina, 19, woman with scabies)

*When all my family members were simultaneously afflicted by the disease, I used to believe it was due to a curse.* (Meseret, 35, woman with scabies)

*When all my family members got scabies, I thought that other people’s jealousy about my life had brought scabies to us.* (Tegenu, 35, man with scabies)

The occurrence of outbreaks was often attributed to demonological causes, and scabies was also sometimes associated with witchcraft (the use of supernatural or magical abilities, rituals, and practices to achieve various ends [35]):

*I stepped over a muart/metet* [bad thing prepared by a person practicing witchcraft after being informed by his or her client to harm others] *on the road to my house*. (Alemitu, 57, woman with scabies)

For some people, scabies was also believed to be caused by an individual’s actions and behaviour—particularly the violation of cultural norms, broken vows made to saints, and failure to observe saint days, which were often perceived as key drivers of the disease.

*One of my daughters had scabies in the past. I took her to St. Michael church for treatment and applied Abune Zarabiruk’s holy water and holy soil all over her body. She was completely cured from the disease and her mother vowed to venerate Abune Zarabiruk’s Day celebrated on 13th of every month. Now I suspect that her mother forgot to observe that specific day and the scabies relapsed on the body of my daughter.* (Embiale, 35, male caregiver)

*.…I should have been attentive in observing Abune Zarabiruk’s day. If I forget and do simple things like cutting branches from trees that is enough to breach the promise. As I was cured from the illness previously through the help of Abune Zarabiruk’s holy soil, I need to refrain from outdoor activities on 13th of every month to remain healthy. Abune Zarabiruk is both merciful and quick tempered. I prayto my God to heal me from this infection.* (Mantegibosh, 55, woman with scabies)

Among some Orthodox Christian followers, there is also a strong belief that the origin of scabies can be traced to evil spirits, a view often linked to stories found in the Bible and other religious texts.

*Scabies is growing in our area because of evil spirits. The evil spirit possessing the body of people made them suffer from scabies. The evil spirit aggravates their pain as it exposed people to the uncontrollable itching and scratching in the name of scabies. Satan and scabies are highly related.... As stated in the bible, Satan tested Job by hitting him with scabies wounds all over his body and tried to erode his trust in God by saying your God let you suffer from this disease…But Job was so strong that he never lost faith on his God. Since then, scabies is associated with Satan and bad sprit.* (Tibebu, 39, male holy water healer)

### 3.3. Traditional healing for scabies control

#### 3.3.1. ‘Holy water’.

Holy water, *tsebel* in Amharic, is an Ethiopian Orthodox Christian curative system believed to embody the spiritual healing power [36,37]. It is widely used in Debre Elias, and it is used for the treatment of various conditions:

*Holy water is my choice. It is the ultimate source of cure for bad things in life. I first try to cure my health problems using holy water. Holy water is part of my life, and it is very convenient for us to use it.* (Gashew, 40, man without scabies)

During field observations in Dejiba *kebele*, some people showing symptoms of scabies were using holy water to treat their condition. The first author also had the opportunity to accompany Health Extension Workers (HEWs) during a house-to-house health intervention. During polio vaccination visits, one HEW identified two individuals with scabies who were receiving treatment through holy water and had not accessed biomedical care. The HEW advised them to attend the local health centre for treatment, although both expressed doubts that they would do so. In another household, a woman refused to allow her one-year-old son to be vaccinated against polio: “My child is receiving prayers from the Orthodox Church and the vaccine may clash with it”, she said. These observations illustrate the challenges frontline health workers face in promoting biomedical interventions and the continued importance of alternative health beliefs and practices within the community.

Field observations indicated that the use of holy water for scabies treatment commonly takes place in holy water sites, usually located in Orthodox churches. Holy water may also be taken home and used there, particularly when scabies is attributed to witchcraft or spiritual forces, reflecting the close intersection of spiritual beliefs and healthcare practices in the area. For Orthodox Christians, holy water is believed to possess God’s healing power. Those seeking holy water treatment often travel to holy water sites, where they sprinkle or wash their bodies with the water. Alternatively, holy water is transported home in bottles or jerrycans and applied by family members, or a priest may be invited to pray for and baptise affected individuals in their homes. These practices can occur throughout the year in response to symptoms of illness. In Debre Elias, however, major saints’ feast days are particularly important occasions, during which worshippers receive holy water blessings that are believed to promote healing and protect against disease. Following these ceremonies, worshippers frequently carry bottles and jerrycans of holy water to friends and relatives who are unable to attend, especially those who are bedridden or otherwise unwell.

When asked, several informants expressed a preference for holy water treatment, citing their belief in its spiritual and therapeutic efficacy: *Holy water is the best treatment given from God.* (Bogalech, 30-year-old female care giver)

Holy water is freely available and accessible to everyone in Debre Elias. A strong sense of belonging and ownership toward holy water sites, along with a deep historical and spiritual attachment to the practice, makes it a primary healing option for scabies and other health conditions. During observations and interviews it became very clear that several factors contribute to its popularity, including the geographical proximity of holy water sources, the absence of service fees, the perception that traditional healers offer more holistic care than biomedical professionals, and limited access to biomedical healthcare services. Additionally, social referral—recommendations and shared experiences within the community—play a significant role in shaping individuals’ treatment-seeking decisions:

*Some of my relatives and neighbors urged me to use Abune Zarabiruk’s holy water and holy soil witnessing that for wounds and skin disorders it is the best healing.* (Alemitu, 57, woman with scabies)

Health workers in Debre Elias are well aware that holy water is very often the first line of treatment for scabies:

*… my parents got scabies during the 2021 outbreak. I told them to go to the* [biomedical] *health centre immediately, but they ignored my advice and went to the holy water. Then when the symptoms were not disappearing and were getting worse, they visited the* [biomedical] *health facilities.* (Wubirst, 30, female nurse)

… *when I asked people with scabies about their treatment history, most of them say that they’ve tried to treat scabies with holy water. They believe that scabies is caused by spiritual forces, and they visit faith-based healing practices before they come to the* [biomedical] *health centre. They come to us when the holy water treatment is not successful.* (Melese, 30, male nurse)

#### 3.3.2. Herbal scabies treatments.

Herbal treatment is another widely used traditional approach to scabies treatment in Debre Elias. In most circumstances, traditional herbal healers acquired the *wisdom of healing (*as is locally referred to, in Amharic: *yemefewes tibeb*), by observing the traditional healing practice being carried out by senior healers and consulting them when in doubt:

*Herbal medicine prepared by mixing eret* [Aloe pulcherrima], *birbira* [Millettia ferruginea] *and lemon ingredients would be applied over the skin of the affected person and exposed to the sunlight for thirty minutes for seven days. The disease will vanish at the end. The medicine prepared from these medicinal plants will completely cure scabies.* (Geremew, 40, male traditional herbal healer)

Most of the interviewed herbalists indicated that scabies can be effectively treated using traditional herbal medicine. Herbalists described two types of scabies based on the salient symptoms. The first one, referred to in Amharic as *abash or aflegna ekek* (‘active scabies’), was known for its visible skin rashes and lesions in different parts of the body. The second type, called *denkoro ekek* in Amharic (‘dormant scabies’), is used to refer to the recent scabies outbreaks, and is known to make smaller ashy rashes, lesions, and wounds than the first type. According to participants, itching is more severe in the second category.

*Abash ekek* [active scabies] *presents with numerous lesions and rashes.* (Tibebu, 39, male traditional herbal healer)

*There are no visible lesions or complicated wounds in denkoro ekek* [dormant scabies]*, but it changes the color of the skin to ashy and the itching is always there.* (Solomon, 30, male traditional herbal healer)

This classification of scabies indicates that traditional herbal healers are very much familiar with the disease and its clinical manifestations. Herbal healers diagnose scabies by examining skin lesions and asking affected individuals about the nature and severity of itching, as well as other associated symptoms. Once a diagnosis has been made, healers employ a range of medicinal plants to treat scabies. During interviews and observations, *endod* [Phytolacca dodecandra, commonly known as ‘African soapberry’] was the most frequently mentioned herbal treatment for scabies. The herbal preparations are typically used as ointments on the lesions and affected body parts. In contrast, biomedical science categorizes scabies in terms of the number of mites found in the skin, using the term ‘classic’ when there are only few mites in the skin and ‘crusted’ when there is abundance of highly contagious mites, which may become chronic and, in severe cases, life-threatening [38].

Fieldwork observations also revealed that traditional herbal healers actively advertise and promote their services through roadside signboards displaying the name of the traditional treatment centers and the list of diseases they treat. It is common to see *ekek* (the Amharic name for scabies) listed alongside other diseases on these sideboards. The prominent inclusion of scabies in such advertisements suggests that traditional healers recognise the disease as an important health concern in the area and actively market their herbal treatments as a means of addressing it. This visibility also reflects the established role of traditional healing centres in local healthcare-seeking practices and the continuing demand for their services.

Traditional etiological beliefs about scabies strongly influence treatment choices (Table 1): Holy water is commonly used to address perceived supernatural or personalistic causes of the disease, while herbal medicine is employed to treat the physical symptoms—such as lesions and wounds—believed to result from ecological or environmental factors. The following table summarizes the complexity of the perceived causes and traditional treatments of scabies in Debre Elias.

**Table 1:** Summary of perceived aetiologies of scabies and their respective traditional treatments.

Perceived causes of scabies in Debre Elias	Specific Examples	Most used Treatment
**Supernaturalistic-personalistic factors**	Curse	Holy water
	Witchcraft	
	Demonological cause/evil spirit	
	Punishment from God	
	Failure to observe Saint days (e.g., Abune Zarabiruk)	
	Forgetting ritual	
**Naturalistic-Ecological factors**	Worms	Herbal medicines
	Fly	
	Insect bite	
	*Mich* (caused by exposure of sweating body to sunlight)	
	*Webek (*when people lay down on the grazing land and the contact between their body and the land heated by the sun creates unwanted humidity)	
	Moving to a new house	

As Table 1 shows, when scabies is believed to be caused by evil forces, holy water is the preferred treatment, as it is thought to not only alleviate the scabies symptoms but also cleanse individuals from sins and dispel any bad spirits believed to manifest through these symptoms. On the other hand, traditional herbs are predominantly used when scabies is attributed to ecological factors, with traditional herbal healers focusing on treating the lesions and wounds associated with scabies. Importantly, these categories are not mutually exclusive: individuals with varying beliefs about the cause of scabies may seek treatment from different traditional sources.

Participants identified several factors that encouraged the use of traditional scabies treatments, including the cultural competence and accessibility of traditional healers, limited access to biomedical services, work demands and time constraints, the distance to health facilities, concerns and hesitancy regarding biomedical treatment, and negative experiences with some biomedical health workers. Together, these factors shaped treatment-seeking decisions and contributed to the continued reliance on traditional healing systems.

### 3.4. Biomedical treatments for scabies in Debre Elias

According to interviewed local health workers, there are three complementary biomedical strategies employed to control the scabies in Debre Elias: case management, contact management, and environmental management. Case management involves the diagnosis and treatment of individuals presenting with scabies symptoms. Contact management entails treating the close contacts of diagnosed individuals even when they do not exhibit symptoms, in order to interrupt transmission. Environmental management focuses on reducing the risk of transmission through contaminated clothing, bedding, and other household materials that may harbour scabies mites.. In practice, this approach is implemented through water, sanitation and hygiene (WASH) interventions, including washing, disinfecting, and improving the handling of items used by infected individuals.

For case management—the focus of this paper—biomedical healthcare providers in Debre Elias treat scabies using both oral and topical scabicides. Fieldwork observations revealed that the most commonly used biomedical treatments were benzyl benzoate lotion (BBL) and sulfur lotion. This is primarily because the two other first-line scabies treatments are prohibitively expensive for the Ethiopian government to procure and are typically only available through external donations. One health worker acknowledged the challenges associated with scabies treatment stating:

*Benzyl benzoate lotion and sulfur are the common scabies treatments prescribed in our health centre. These two are taken as a form of lotion and they are effective if administered correctly. Permethrin is the first line scabies treatment, but most of the time is not available in our drug store.* (Assefa, 25, male nurse)

Perhaps for this reason, in Debre Elias biomedical treatments coexist alongside traditional treatments, and it is often the case that people try first the traditional treatment options before deciding whether to try biomedical ones. For instance, Setegn had Community-Based Health Insurance (CBHI) introduced by the Ethiopian government in 2011 to increase primary health care coverage in exchange for a small annual contribution. Holding the CBHI card was key for Setegn and his children to be able to receive free scabies treatment in the health centre. Having access to free treatment did however not change the fact that, like many others in Debre Elias, Setegn did not seek biomedical treatment until seeing that the symptoms persisted after trying home remedies.

*We had been waiting for a long time before deciding to come to the* [biomedical] *health center hoping that we would get well within a few days. We decided to visit the health center when the illness* [scabies] *prevented us from doing our everyday activities. My children have been suffering from ekek* [scabies] *for over a month. It wasn’t this bad earlier, and I was telling them to wash their clothes and their mother tried to administer home remedies to control the disease initially. But I brought them now as the lesions have spread all over their body parts and they are unable to urinate because the disease has heavily wounded their private parts.* (Setegn, 55, man with scabies)

Among participants who had sought biomedical treatment, several reported that they had previously tried traditional remedies before turning to biomedical care when these treatments were perceived to be ineffective:

*Before I received treatment from the* [biomedical] *health centre, I tried to control the severe itching with holy water and emnet* [holy ash or soil]. (Tegenu, 35, man with scabies)

According to both biomedical health practitioners and people who had had scabies, this “wait and see” attitude of people with scabies often means that when people visit the biomedical health systems the scabies is harder to treat, as they are often having severe wounds and lesions.

#### 3.4.1. Determinants of, and challenges to, biomedical treatment-seeking.

Since the 2015 scabies outbreak in Amhara, only trained nurses and HEWs are authorised to diagnose and treat scabies within the public health system. Debre Elias has no dermatologists and limited diagnostic resources, and diagnosis is based on visual assessment of symptoms, consistent with WHO recommendations.

The availability of health extension workers (HEWs) and community-based health insurance (CBHI) scheme are important determinants of healthcare access and people’s health itineraries in Debre Elias. Through household visits, disease surveillance activities, health education campaigns, and follow-up support for affected individuals, they play a critical role in identifying scabies cases and linking community members to biomedical care. The follow-up support that HEWs provide to people affected by scabies was widely appreciated by participants in Debre Elias and contributed to positive perceptions of the biomedical health system:

*We go to the health post seeking advice from the health extension workers… they also visit us to see how well we are doing in cleaning our body and clothes.* (Meseret, 35, woman with scabies)

*The close relationship we establish with the people in the village eases the process of accessing healthcare and improves health service utilization.* (Sewunet, 29, female HEW)

Community-Based Health Insurance (CBHI) is necessary for people to access healthcare services in public biomedical facilities in Debre Elias, but coverage is not universal. The main reason for this is that CBHI enrolment is organised at the household rather than the individual level. As a result,.newly married couples, divorced individuals, and religious students often lack access to CBHI benefits and face unaffordable out-of-pocket healthcare costs. Consequently, many rely on home remedies or comparatively affordable alternatives such as holy water to manage scabies symptoms. Even among those enrolled in CBHI, timely treatment is not always guaranteed, as essential medicines may be unavailable at local health facilities:

*I am beneficiary of community-based health insurance but we were told that the drug is unavailable …. They gave us prescriptions to buy medicine from the private drug stores. All medicines should be made freely available.* (Tegenu, 35, man with scabies)

*I went to the health centre but the medicine was not available… the health workers told me to come another time when the medicine arrives and then I was applying the holy water. I heard from the health workers that the medicine had arrived and went for a second time.* (Wubalem, 30, woman with scabies)

Shortage of medications poses important constraints on scabies control. Participants and health officials described frequent shortages of essential medicines as a major barrier to timely and effective treatment. According to several health officials, no dedicated government budget is allocated for the procurement of first-line scabies medications because these treatments are not included in the national essential medicines list. As a result, health facilities often experience stock-outs, limiting their capacity to provide adequate care and contributing to delays in treatment. These structural challenges also undermine confidence in biomedical services and encourage people to seek alternative treatment options, including home-based and herbal healing..

*The lack of adequate medications truly impacted the success of the intervention. The lack of medical supplies is one of the reasons for the continued scabies prevalence.* (Adugna, 35, male health expert)

During fieldwork, various informants admitted that medicine shortage in the health centers often decreased people’s eagerness to seek biomedical health advice. **I**nterviews with health workers further suggest that treatment rates are low in Debre Elias. In the absence of the first line scabicides, BBL and sulfur are widely used, but these treatments have bad smell, which contributed to high treatment dropouts.

*After applying the treatment for three days, my son was not happy with the bad smell of the drug. He threw away his blanket and it is still hanging outside the house.* (Bekele, 54, male care giver)

Interviewed health workers reported that they typically advised patients to tolerate the smell and continue using the medication until they had fully recovered. However, field observations indicated that biomedical health providers did not always explain how prescribed acaricides should be used, or the secondary effects. In some cases, treatments were dispensed with little or no demonstration of their proper application, creating a communication gap between healthcare providers and patients. Difficulties in recognizing scabies as a serious health condition, coupled with distrust and fear of biomedical treatments, contributed to treatment refusal and non-adherence. Low health literacy and inadequate communication regarding treatment use appeared to reinforce these concerns. Furthermore, when available, medicine labels and prescriptions were typically written in English, a language that many rural residents could not read, creating additional barriers to understanding and correctly using prescribed treatments.

*I didn’t have enough information about the medicine, and I feared that it might have a chemical that does not go away when I wash my hands… I chose to endure the pain rather than spoil my tella* [the local beer] by contaminating with the chemical. (Banchamlak, 40, woman with scabies)

### 3.5. Tensions and power imbalances between biomedical and traditional approaches to scabies management

Biomedicine is often the last resort in Debre Elias. As shown, for many reasons people commonly seek treatment via self-medications and traditional healing before trying biomedical care. Informal conversations conducted during field observations revealed that even those who seek biomedical treatment also sometimes return to traditional treatments. This was acknowledged by several participants, including traditional healers and biomedical healthcare providers. However, this therapeutic movement between systems was viewed by biomedical health workers as a significant concern, as they worried that low and delayed biomedical treatment uptake contributes to continued transmission of scabies and complicates disease management:

*People with scabies only come to the biomedical treatment centre when their condition becomes critical. In the meantime, they could spread the disease to several people.* (Melese, 30, male nurse)

Several biomedical health workers attributed low treatment rates and delayed biomedical health-seeking among people with scabies to the widespread use of traditional medicine. During interviews, it became clear that many biomedical providers held negative perceptions of traditional healing, often dismissing it as irrelevant to public health. Fieldwork observations further revealed that health workers frequently challenged and attempted to discredit traditional practices during patient encounters. When asked about the effectiveness of traditional healing for treating scabies, one health expert responded:

*… never! it is not helpful. We are facing challenges because of it. When people are in trouble, they absurdly used whatever solutions by themselves including medicinal plants. All these attempts could not kill the mite. Only the biomedical treatments cure people from scabies and I do not think traditional medicine is the solution for scabies.* (Adugna, 35, male health expert)

Most interviewed health workers conceptualized traditional medicine in a very demeaning manner, and diminished its role in the treatment of scabies. A 34-year-old HEW even labelled traditional medicine as ‘*bead neger’* (roughly translated as ‘noxious’). As part of the belittling of traditional medicine by some biomedical health workers, practitioners of traditional herbal medicine were sometimes referred to as *kitel betash* (‘leaf cutter’), a derogatory term that reflects occupational stigma and the subordinate position assigned to traditional practitioners within local hierarchies of medical legitimacy. This was despite, as illustrated in the above sections; most people in Debre Elias initially seek treatment via traditional medicine.

Observations evidenced that some biomedical health workers even ridiculed patients who reported having visited herbal traditional medicine practitioners, referring to this it as a backward practice. In turn, some traditional healers also displayed a dismissive attitude towards biomedical practices, although not to the extent exhibited by some biomedical health workers. Perhaps because of this hostile relationship, there is no referral system from the biomedical treatment facilities to traditional medicine centres to scabies treatment. Similarly, for some holy water healers their patients should either choose holy water treatment or biomedical one:

*When people come to the holy water centre, they are not allowed to bring any biomedical treatments. They should stick to either of the treatments at a time. Not only scabies affected people but no one taking the modern medicines for any form of disease will not be entertained in the holy water treatment centre. We have orientation session for newcomers and all the required behaviours they need to follow while staying in the holy water treatment centre would be communicated on their arrival in the centre.* (Tibebu, 39, male holy water healer)

These tensions between biomedical healthcare providers and traditional healing practitioners and their followers have contributed to the existence of a fragmented health service system. This fragmentation has, in turn, impacted the treatment-seeking behaviour and adherence to treatments among individuals with scabies in Debre Elias. The lack of cooperation between the two healthcare systems exacerbates the health issues of the population, as individuals are stretched to seek treatment from both systems. The tension also arises from the hegemony of biomedical scabies control initiatives coexisting with deep-rooted cultural acceptance of traditional healing practices.

## 4. Discussion

Both traditional and biomedical scabies control strategies coexist informally in Debre Elias. Pluralistic disease management occurs when people use both traditional and biomedical scabies treatments, sometimes sequentially, some other times simultaneously [39]. As shown, despite only biomedical approaches to scabies control are currently recognised in Ethiopia, people with scabies usually start their scabies treatment journey by seeking help from the traditional treatment centres and often ask the advice from the biomedical health professionals if and when they feel that their symptoms did not improve. Often, they also return to traditional healing specialists after receiving biomedical treatment. These are commonly reported health-seeking behaviours across contexts and skin NTDs. For instance, a study conducted in South-eastern Ethiopia also reported that people with scabies sought treatment in biomedical health facilities when the symptoms become complicated or they get re-infected [40]. Similarly, a study conducted on podoconiosis in Debre Elias and other five districts of the Amhara region reported that people’s belief in heredity or divine punishment as a cause of the disease led them to seek treatment from traditional sources and often delayed to visit biomedical facilities [41]. Pérez *et al*, and Elsheikh *et al*. also reported very similar health itineraries in the case of cutaneous leishmaniasis in Guatemala [42,43] and mycetoma in Sudan [44]. In line with these studies, our research demonstrates that people do not exclusively choose one health system over the other; rather, they navigate between traditional and biomedical treatments, sometimes moving back and forth. This highlights the need to develop a formal pluricultural health management system, and strengthen collaborations between these coexistent systems. The need to explore ways to synergise various medical systems and their implications in shaping people’s health seeking behaviours has long been well documented in medical anthropology, and these insights should play a crucial role in designing appropriate disease control interventions [43–46]

We have shown that people attributed scabies outbreaks to various supernaturalistic and personalistic causes, with epidemics and scabies cases being perceived as manifestations of God’s anger and punishments for wrongdoings [47]. Consequently, holy water and herbal oitnments are the common traditional treatments used by people with scabies in Debre Elias. Ethiopian Orthodox holy water sites attract large numbers of visitors seeking treatment for health concerns including HIV/AIDS, demonic afflictions [28], mental illness, minor social difficulties [48], and skin conditions such as scabies [49]. As shown, in Debre Elias e*ndod* is the preferred traditional herbal treatment of scabies. This aligns with existing research reports that *endod* is the most frequently used herbal treatment out of the 52 medicinal plants in Ethiopia [19]. The existing published literature also mentions *qechemo* (phyllanthus ovalifolius) [50], *limich* (clausena anisate) [51], *chifrig* (sida massaica) and *dedeho* (euclea racemose) [52] as the common herbs used to treat scabies in Amhara region and Debre Elias.

The shortcomings of the biomedical scabies treatment approaches, including the lack of medical supplies, also influences the treatment-seeking behaviours and itineraries. In line with these findings, a recent study conducted in Southern Ethiopia also revealed that traditional medicine is widely recognized as a legitimate medical practice among many individuals and is expected to maintain its acceptance, even amidst the growing influence of biomedicine [53].

Despite their informal co-existence, traditional and biomedical practitioners have never attempted to work collaboratively to jointly advance the control of scabies in Debre Elias. However, our findings suggest that the collaboration of traditional medicine and biomedicine practitioners could bring a vital step to halt the persisting scabies occurrence in Debre Elias. This is also supported by existing research findings in various other contexts and health issues, from occupational injuries to other skin NTDs in Africa and Latin America [44,45,54]. For instance, Baheretibeb *et al*. concluded that cross-system collaborations are indeed a feasible and potentially valuable approach to address biomedical resource constraints and psychiatric ill-health in Ethiopia [37]. Mutual recognition is a prerequisite for the success of the collaboration. However, the existing health policy frameworks in Ethiopia do not encourage recognition nor collaborations between traditional and biomedical health systems, and they often use the norms of the latter to regulate the former. Our research indicates that this obstructs scabies control in that it fosters tensions and competitions among care seekers and treatment providers alike.

There is however a notable lack of government initiatives aimed at strengthening collaboration between traditional healers and the formal public healthcare system in Debre Elias and Ethiopia more generally [55]. The secretive working culture of traditional healers, combined with limited governmental recognition and support, in turn hinders the advancement of traditional medicine. Herbalists commonly hide their knowledge regarding the collection, preparation, and administration of medicines from the public, clients, and other healers as a professional secret. They are suspicious of people, particularly government officials and biomedical health providers. At the same time, frequently face challenges in effectively managing conditions such as scabies, even when treatment options are available. As shown, these dynamics contribute to a cycle of mistrust, low treatment uptake, high dropout rates, and recurrent outbreaks. This paper therefore argues for the adoption of a collaborative pluralistic approach that recognizes the coexistence and value of both traditional and biomedical systems of healing involving the mutual recognition of the co-existing systems of healing. Developing respectful partnerships between traditional healers and biomedical health providers should be considered a key strategy for improving scabies control in Debre Elias. Initial efforts could focus on educational and sensitization programmes for practitioners from both systems [53], alongside the establishment of formal mechanisms for mutual referral and cooperation [44].

## 5. Study limitations

We did not assess the effectiveness of traditional healing methods in controlling scabies symptoms, and more research is needed to establish conclusive evidence in this regard. Another potential limitation is related to the nature of scabies symptoms and the common diagnostic methods used to identify scabies in Debre Elias district. People with scabies included in this study were those who had learned of their condition through either a suspected or clinical diagnosis, and, admittedly, many lived without confirmed diagnosis. Consequently, some of the research participants may have had symptoms (e.g., itching and skin lesions) that only resembled scabies. Our study nevertheless brings important insights into people’s health management, including scabies.

In our study we focused on scabies treatments and interventions provided by healthcare workers in the formal biomedical facilities. However, there are also informal biomedical actors who sell treatments for scabies without prescriptions (e.g., local drug stores). We did not include these informal biomedical scabies treatment providers in this study because these dynamics were found midway through data collection after securing ethical clearance for the fieldwork. Future research may investigate the implications of informal biomedical actors in the control of scabies in Debre Elias.

## 6. Conclusions

The large-scale scabies outbreak in the Amhara region in 2015 marked a significant turning point in Ethiopia’s response to scabies, establishing biomedical treatment as the mainstream approach to scabies control. Our research however shows that, in Debre Elias, biomedical and traditional healing systems continue to coexist, with individuals frequently drawing on both forms of care either sequentially or simultaneously. Common traditional approaches include faith-based healing practices such as the use of holy water, holy soil or ash, prayer, as well as herbal remedies.

Our study further reveals that the limited recognition of traditional healing practices within the formal health system, coupled with weak collaboration between traditional healers and biomedical practitioners, undermines the effectiveness of scabies control efforts. This is partially influenced by mutual disregard and reject that often negatively impacts on the relations biomedical health professionals have with those seeking help for their health issues. Mutual distrust and the absence of positive engagement often discourage open communication, contribute to fragmented care pathways, and affect treatment uptake and adherence, ultimately also affected scabies prevalence. As shown, in this context where multiple health belief systems coexist, disregarding traditional healing practices risks overlooking important factors that shape health-seeking behaviour and community responses to disease.

These findings suggest that a collaborative pluralistic approach offers considerable potential for improving scabies control. By fostering mutual recognition and respectful partnerships between traditional healers and biomedical providers, such an approach could strengthen trust, enhance community engagement, improve treatment accessibility and utilization, and promote earlier and more sustained healthcare seeking. Importantly, collaboration may also reduce treatment delays, lower dropout rates, and contribute to more effective and culturally acceptable interventions.

Rather than viewing medical pluralism as a barrier, policymakers and health practitioners should recognize it as a potential resource for strengthening disease control efforts. Greater government support for dialogue, joint training and sensitization activities, and the development of formal referral mechanisms between traditional and biomedical practitioners could provide important starting points. Future research should explore the most effective models of collaboration and evaluate their impact on scabies prevention and treatment outcomes. Ultimately, the co-production of strategies involving traditional healers, biomedical providers, policymakers, and community members will be essential for developing contextually appropriate, culturally responsive, and sustainable approaches to scabies control in Debre Elias and Ethiopia more broadly.

## Supporting information

S1 File Interview Guide. (DOC)
