# Supplementary material for: From co-existence to collaboration: The need to move towards an inclusive (pluricultural) scabies management approach in Debre Elias (Ethiopia)
Source: PLoS Negl Trop Dis. 2026 Oct 14;20(10):e0014773. doi: 10.1371/journal.pntd.0014773 (PMC13667770; doi:10.1371/journal.pntd.0014773)
Supplement: Supplementary file 1 [file pntd.0014773.s001.doc]

**Appendix: Interview Guide**

I.	**People with scabies**

Interviewer ________Date _______Start time ____End time ___Interviewee Code__

Background Information: Demographic and socio-economic characteristics of participants

1.	Age

2.	Gender

3.	Occupation

4.	Marital status

5.	Educational status/level

6.	Religion/ religious group

7.	Household size /number of dependents of informant

8.	Can you please tell me how much money you earn per day (or week/month)?

9.	Do you have any additional income or support from relatives and family members? If yes, how much? How often?

10.	Tell us about your living conditions.

Probe: How many people live in this house?

11.	Do you have access to water? How do you access water? How many litters/jerrycans do you need? Do you think the water available is wholesome and sufficient?

12.	Do you have access to shower and hygiene facilities?

Probe: Tell us about the washing experience in the household and how often you wash your cloths and body?

Probe: Is your washing experience influenced by your access to water?

13.	Do you have community-based health insurance card? Was it easy to get?

14.	Do you regularly see the doctor or nurse? Why? Why not? For what health problems if you do not mind me asking?

15.	Do you regularly see the traditional healers? Why? What not? For what health problems if you do not mind me asking?

16.	How do you choose between traditional healers and extension workers? Which one you prefer and why?

17.	Have you ever been diagnosed with scabies? When?

18.	How many times you have had scabies?

19.	Who else in your family has had it?

Impacts: Everyday life of people living with scabies

1.	What do you normally do during the day? What are the common activities you accomplish in your everyday life?

2.	Do you feel scabies has been affecting your daily life? In what ways?

3.	Has it affected your earnings? How?

4.	Has it affected your relationships with other people in your district, in what ways? Why do you think this is?

5.	Can you name some activities you were doing well before you had scabies but that cannot do now?

6.	How do those in your social environment (family, neighbors, friends and others) react when they see your scabies lesions? Have they behaved any differently with you or your family? In what ways? Why do you feel this is?

7.	Is there anything you feel you could do to prevent these impacts on your life and that of your family??

8.	Do you feel supported by your family or health professionals? Why? Why not? In what ways?

9.	What else you feel could be done to help cure and prevent the impacts of this disease on affected people?

Scabies, health itineraries, coping and management

1.	How did you start noticing something was happening? (How you first realized the symptoms of your illness?)

2.	How does it affect you? What do you feel in your body?

3.	How did you learn that you are infected with scabies? Who told you?

4.	Do you know what caused it? (prompt them to explain the causes as they understand them)

5.	Could anything have been done to prevent this from happening? What? Did you try to prevent it? How? Why did you take these measures and not others?

6.	What are you doing to control it?

7.	Do you think it can be cured? How?

8.	What are you doing to cure it?

9.	Where did you learn about this?

10.	Have you seen the health extension worker or healer about this?

11.	How long did you take to see them since you felt something was wrong?

12.	Did you try to sort it by yourself before seeking health? Why?

13.	What did you do to try to sort it? Who advised you?

14.	What is your opinion of the treatment strategies conducted to treat scabies in the district?

15.	Is there anything else that you feel is important but I have not asked you?

Thank you for taking part in this interview!

**II.	People without scabies**

Interviewer ________Date _______Start time ____End time ___Interviewee Code__

Background Information: Demographic and socio-economic characteristics of participants

1.	Age

2.	Gender

3.	Occupation

4.	Marital status

5.	Educational status/level

6.	Religion/religious group

7.	Can you please tell me how much you earn per day (or week/month)?

8.	Do you have any additional income or support from relatives and family members? If yes, how much?

9.	Do you have access to water? How do you access water?

10.	Do you regularly see the doctor or nurse? Why? Why not? For what health problems if you do not mind me asking?

11.	Do you regularly see the traditional healers? Why? What not? For what health problems if you do not mind me asking?

12.	How do you choose between traditional healers and extension workers? Which one you prefer and why?

Impacts in everyday life, understandings, management and influencing factors

13.	What do you normally do during the day? What are the common activities you accomplish in your everyday life?

14.	How do you feel scabies impacts on people?

15.	Do you feel the fact that there is people here with scabies has affected your daily life? In what ways?

16.	Do you have friends/relatives who had or has scabies?

17.	Did the fact that they had scabies changed your relationships? How did the relationship change? Why do you think this happened?

18.	Has it affected your relationships with other people in your district, in what ways? Why do you think this is?

19.	Have you ever been diagnosed with scabies? When?

20.	How many times you have had scabies?

21.	Who else in your family has had it?

22.	Can you name some activities you were doing well before you had scabies but that cannot do now?

23.	How do you feel when others see your scabies lesions? Do you behave any differently with people when they have scabies? In what ways? Why do you feel this is?

24.	Do you know what causes scabies? (Prompt them to explain the causes as they understand them)

Probe: why do you think people are affected by the disease?

25.	Who are more prone to the disease in your locality?

26.	How, according to you, are people with scabies treated in this community? Why do you think this is? Do you treat people with scabies any differently? Why? Why not?

27.	What measures you practiced to keep yourself and family free from scabies?

28.	What do you think should be done to control the incident of scabies?

29.	Is there anything you feel you could do to prevent you or your loved ones from getting scabies? What?

30.	What else you feel could be done to help cure and prevent this disease?

31.	Could you tell me anything you have been done to prevent this from happening? What? What do you do to avoid getting this disease? Why did you take these measures and not others?

32.	Do you think it can be cured? How?

33.	Where did you learn about this?

34.	What is your opinion of the treatment strategies conducted to treat scabies in the district?

Thank you for taking part in this interview!

**III.	Healthcare Professionals**

Interviewer ________Date _______Start time ____End time ___Interviewee Code__

Background Information: Demographic and socio-economic characteristics of participants

1.	Age

2.	Gender

3.	Occupation

4.	Marital status

5.	Educational status/level

6.	Tell us about your position in this health care center?

7.	What are some of your roles and responsibilities in the center?

8.	How long have you been working here?

Scabies related questions

9.	Why do you think scabies is common here?

Probe: What do you think are the fundamental causes of scabies among the community?

10.	From your observation and experience who are the most affected individuals (in terms of age, gender and other status)?

Probe: Why do you think they are more affected than others?

11.	Do the affected individuals seek the medical attention of healthcare professionals? Why? Why not? Why do you think this is?

12.	Do they normally seek traditional treatment before going to the health center? Why do you think this is?

Probe (if yes): Does it affect treatment procedures? In what ways?

13.	In your experience, what causes scabies? Why do you think this causes scabies?

14.	How can it be prevented? Why do you think this can help prevention?

15.	What are the best treatment options? Why do you think this the best option?

16.	Do you feel traditional medicine is helpful in preventing/treating scabies? Why? Why not?

17.	Do you think people in the community understands scabies differently? Why? Why not? In what ways?

18.	What scabies prevention and control strategies are implemented in your institution? Probe: Do this work well? Why? Why not?

19.	Do you feel scabies impacts the social relations in the community? In what ways? Why do you feel this is?

20.	Is there anything else that could be done in your experience to improve scabies prevention and control here? What? Why do you feel this could help?

21.	Do you have something to add?

Thank you for your participation!!

**IV.	Traditional Healers**

Interviewer ________Date _______Start time ____End time ___Interviewee Code__

Background Information: Demographic and socio-economic characteristics of participants

1.	Age

2.	Gender

3.	Occupation

4.	Marital status

5.	Educational status/level

6.	Is providing traditional healing your full-time job?

7.	What are your roles and responsibilities as traditional healer?

8.	How long have you been providing traditional healing?

9.	How did you learn this?

Scabies related questions

10.	Do you think scabies is common here? Why may this be?

Probe: What do you think are the fundamental causes of scabies among the community?

11.	From your observation and experience who are the most affected individuals?

12.	Why do you think they are more affected than other?

13.	Do the affected individuals seek the medical attention of healthcare professionals? Why? Why not? Why do you think this is?

14.	Do they normally seek traditional healing treatment? Why do you think this is?

15.	In your experience, what causes scabies? Why do you think this causes scabies?

16.	How can it be prevented? Why do you think this can help prevention?

17.	What are the best treatment options? Why do you think this is the best option?

18.	Do you feel formal biomedical medicine is helpful in preventing/treating scabies? Why? Why not?

19.	What scabies prevention and control strategies are implemented the region? Do you feel these work? Why? Why not?

20.	Have you ever collaborated with healthcare workers? When? Why/why not? How did this work?

21.	Do you feel scabies impacts the social relations in the community? In what ways? Why do you fell this is?

22.	Is there anything else that could be done in your experience to improve scabies prevention and control here? What? Why do you feel this could help?

23.	Do you have something to add?

Thank you for your cooperation!

V.	**Government Officials including Ministry of health officials**

Interviewer ________Date _______Start time ____End time ___Interviewee Code__

Background Information: Demographic and socio-economic characteristics of participants

1.	Age

2.	Gender

3.	Occupation

4.	Marital status

5.	Educational status/level

6.	Tell us about your position in this office?

7.	What are some of your roles and responsibilities?

8.	How long have you been working here?

Scabies related questions

9.	Why do you think scabies is common in some regions of the country? In what regions exactly is it more common?

10.	Who are the most affected individuals?

11.	Why do you think they are more affected than other?

12.	What do you think causes of scabies?

13.	How can it be prevented? Why do you think this can help prevention?

14.	What are the best treatment options? Why do you think this is the best option?

15.	Do you feel traditional medicine is helpful in preventing/treating scabies? Why? Why not?

16.	What scabies prevention and control strategies are implemented by your institution? Since when are these running?

Probe: do these work well? Why? Why not? What in your experience works well and does not?

Probe: Do you involve the local communities or traditional healers in the implementation of these control plans/ strategies? Why? Why not?

17.	Do you feel people affected by scabies are marginalized? Why? Why not? In what ways are they marginalized?

18.	Do you feel scabies impacts the social relations in the community? In what ways? Why do you fell this is?

19.	Is there anything else that could be done in your experience to improve scabies prevention and control here? What? Why do you feel this could help?

20.	Do you have something to add?

Thank you for your participation!!

VI.	**NTD program experts**

Interviewer ________Date _______Start time ____End time ___Interviewee Code__

Background Information: Demographic and socio-economic characteristics of participants

1.	Age

2.	Gender

3.	Occupation

4.	Marital status

5.	Educational status/level

6.	Organization

7.	Tell us about your position in this organization?

8.	What are some of your roles and responsibilities?

9.	How long have you been working here?

Scabies related questions

10.	Why do you think scabies is common in some regions of the country? In what regions exactly is it more common?

11.	Who are the most affected individuals?

12.	Why do you think they are more affected than other?

13.	What do you think causes of scabies?

14.	How can it be prevented? Why do you think this can help prevention?

15.	What are the best treatment options? Why do you think this is the best option?

16.	Do you feel traditional medicine is helpful in preventing/treating scabies? Why? Why not?

17.	What scabies prevention and control strategies are implemented by your institution? Since when are these running?

Probe: do this work well? Why? Why not? What in your experience works well and does not?

Probe: Do you involve the local communities or traditional healers in the implementation of these control plans/ strategies? Why? Why not?

18.	Do you feel people affected by scabies are marginalized? Why? Why not? In what ways are they marginalized?

19.	Do you feel scabies impacts the social relations in the community? In what ways? Why do you fell this is?

20.	Is there anything else that could be done in your experience to improve scabies prevention and control here? What? Why do you feel this could help?

21.	Do you have something to add?

Thank you for your participation!!
